# MOLUNGN: a multi-omics graph neural network for biomarker discovery and accurate lung cancer classification

**DOI:** 10.3389/fgene.2025.1610284

**Published:** 2025-06-04

**Authors:** Daifeng Zhang, Guoqiang Bian, Yuanbin Zhang, Jiadong Xie, Chenjun Hu

**Affiliations:** ^1^ School of Artificial Intelligence and Information Technology, Nanjing University of Chinese Medicine, Nanjing, China; ^2^ Jiangsu Collaborative Innovation Center of Traditional Chinese Medicine in Prevention and Treatment of Tumor, Nanjing, China; ^3^ Jiangsu Province Engineering Research Center of TCM Intelligence Health Service, Nanjing University of Chinese Medicine, Nanjing, China

**Keywords:** lung cancer, multi-omics data integration, MOLUNGN, GAT, stage prediction

## Abstract

**Introduction:**

Lung cancer continues to pose significant global health burdens due to its high morbidity and mortality. This study aimed to systematically integrate biomedical datasets, particularly incorporating traditional Chinese medicine (TCM)-associated multi-omics data, employing advanced deep-learning methods enhanced by graph attention mechanisms. We sought to investigate molecular mechanisms underlying stage-wise lung cancer progression and identify pivotal stage-specific biomarkers to support precise cancer staging classification.

**Methods:**

We developed a novel multi-omics integrative model, named the Multi-Omics Lung Cancer Graph Network (MOLUNGN), based on Graph Attention Networks (GAT). Clinical datasets of non-small cell lung cancer (NSCLC), including lung adenocarcinoma (LUAD) and lung squamous cell carcinoma (LUSC), were analyzed to create omics-specific feature matrices comprising mRNA expression, miRNA mutation profiles, and DNA methylation data. MOLUNGN incorporated omics-specific GAT modules (OSGAT) combined with a Multi-Omics View Correlation Discovery Network (MOVCDN), effectively capturing intra- and inter-omics correlations. This framework enabled comprehensive classification of clinical cases into precise cancer stages, alongside the extraction of stage-specific biomarkers.

**Results:**

Evaluations utilizing publicly available datasets confirmed MOLUNGN’s superior performance over existing methodologies. On the LUAD dataset, MOLUNGN achieved accuracy (ACC) of 0.84, Recall_weighted of 0.84, F1_weighted of 0.83, and F1_macro of 0.82. On the LUSC dataset, the model further improved, achieving ACC of 0.86, Recall_weighted of 0.86, F1_weighted of 0.85, and F1_macro of 0.84. Notably, critical stage-specific biomarkers with significant biological relevance to lung cancer progression were identified, facilitating robust gene-disease associations.

**Discussion:**

Our findings underscore the efficacy of MOLUNGN as an integrative framework in accurately classifying lung cancer stages and uncovering essential biomarkers. These biomarkers provide deep insights into lung cancer progression mechanisms and represent promising targets for future clinical validation. Integrating these biomarkers into the TCM-target-disease network enriches the understanding of TCM therapeutic potentials, laying a robust foundation for future precision medicine applications.

## 1 Introduction

Lung cancer is one of the leading causes of cancer-related deaths worldwide. Early diagnosis and accurate staging are critical for patient treatment and prognosis. Understanding the progression across different lung cancer stages is essential for elucidating the mechanisms underlying the transformation process of malignant tumors. Various biomarkers emerge during these stage transitions, serving as crucial molecular indicators and nodes within the complex biological networks of cancer progression. By analyzing systems biology data from lung cancer cases, we aim to gain deeper insights into cancer staging. However, current methods for lung cancer staging face limitations in accuracy and comprehensiveness, highlighting the urgent need for novel methodologies to improve precision and reliability.

With the rapid advancement of high-throughput biomedical technologies, researchers now have access to extensive multi-omics datasets, including gene expression profiles, DNA methylation values, and microRNA expression patterns. These datasets offer valuable information sources for disease research but present significant analytical challenges due to their complexity and heterogeneity. Effective integration and analysis of these data are crucial for fully leveraging their potential in translational research.

Since the advent of precision medicine, biomarkers have become increasingly important, widely used in screening, diagnosis, and treatment of major diseases, including viral infections and cancers. The development and application of emerging circulating biomarkers have achieved notable success in targeted drug therapies, cellular therapies, immunotherapies and cancer vaccine development. Compared to traditional medical methods, biomarker-based precision medicine offers substantial advantages in both research and clinical treatment (JIANG et al., 2019). Multi-omics data are characterized by large volumes, diverse data types, and abundant sources. Applying artificial intelligence (AI) techniques to analyze such datasets can significantly facilitate the identification of biomarkers and therapeutic targets for various cancer stages, thereby providing tremendous potential for targeted drug discovery.

Given the high heterogeneity of cancer, identical therapeutic interventions can yield varied outcomes among different patients, necessitating more personalized therapeutic strategies. Precise cancer treatment methods require a profound understanding of tumorigenesis, including genetic mutations, protein alterations, and changes in cancer cell phenotypes. AI technologies, especially deep learning and graph neural networks (GNNs), excel in extracting intricate patterns and internal relationships from genomics, transcriptomics, and proteomics datasets. GNNs, for example, have been successfully employed in predicting protein-ligand affinities and have achieved superior performance in identifying pharmacological inhibitors compared to traditional computational approaches. The study and comprehensive analysis of multi-omics data facilitate the development of personalized medicine for lung cancer treatment. Rapid advances in high-throughput sequencing technologies have made DNA and RNA sequencing more efficient and accessible, generating vast amounts of multi-omics data and enabling molecular analysis. Due to the heterogeneity of cancer and the complexity of biological processes, utilizing multi-omics sequencing data is critical for more accurate cancer classification and tumor analysis. Researchers have proposed various methods to classify cancer types or cluster cell types using multi-omics data (Li et al., 2021; Ma and Zhang, 2019; Zhang et al., 2019; Yang et al., 2019; Wang et al., 2021; Sharifi-Noghabi et al., 2019; Lotfollahi et al., 2022). These studies indicate that using multi-omics data can improve analytical performance and enhance the understanding of key pathophysiological pathways at different molecular levels (Heo et al., 2021). Additionally, AI can identify new biomarkers from data, aiding in tumor screening, detection, diagnosis, treatment, and prognosis prediction, thereby providing optimal treatment for individual patients and improving their clinical outcomes (Liao et al., 2022).

Graph neural networks (GNNs), as an emerging deep learning method, can effectively capture relationships and feature interactions among nodes in complex network structures, showing great potential in the biomedical field. For instance, Mastropietro et al. (2023) proposed using GNNs to predict protein-ligand affinities, capturing complex relationships in molecular structures. Zhi et al. (2021) network pharmacology and GNNs to construct multiple cascaded GNN models, achieving better modeling results and higher accuracy compared to traditional methods, successfully identifying effective inhibitors of dihydroorotate dehydrogenase (DHODH), a target for small cell lung cancer (SCLC), proposing a new drug discovery method. Cao and Gao (2022) proposed GLUE, which is composed of explicitly encoding cross-omics regulatory networks, coupling heterogeneous single-cell data such as scRNA-seq, scATAC-seq and even DNA methylation into a unified latent space in a graph-guided variational framework, and verifying its alignment accuracy and regulatory inference efficiency in a million-scale cell atlas. Xu et al. (2024) proposed the STANDS framework, which maps spatial transcriptomic gene expression, tissue morphological imaging, and control scRNA-seq, among other multi-omics data, to a unified latent space through a GAN-based graph attention and Transformer fusion strategy. This enables the detection of abnormal anatomical domains in multiple samples, cross-sample alignment, and heterogeneous subtype segmentation within a single workflow. Lan et al. (2024) proposed DeepKEGG, which integrates SNV, mRNA, and miRNA omics into a unified latent space through a gene-pathway dual-layer sparse connection and path self-attention mechanism, refreshing the benchmark performance in cancer recurrence prediction and biomarker discovery. The CPathomic model proposed by Li et al. (2025) utilizes cross-modal contrastive learning and an interactive-gate attention mechanism to map the local-global representations of the entire pathological slice and paired genomic vectors to a unified latent space, achieving the complementary fusion of morphological and genomic signals.

In this context, this study proposes MOLUNGN, a novel integrative analytical framework designed to enhance lung cancer staging accuracy through comprehensive analysis of multi-omics data using GNN technology. Approximately 85% of lung cancer patients are diagnosed with lung adenocarcinoma (LUAD) and lung squamous cell carcinoma (LUSC) (Molina et al., 2008), the most common lung cancer subtypes. The TNM staging system, an internationally recognized framework first proposed by Pierre Denoix between 1943 and 1952(Brierley et al., 2017), serves as the foundational principle for lung cancer staging. MOLUNGN constructs a complex network integrating gene-protein-clinical data from lung cancer patients and employs Graph Attention Networks (OSGAT) for feature learning from specific omics data. It further integrates multi-omics data through the Omics-Specific View Correlation Discovery Network (MOVCDN) at a higher-level label space, significantly enhancing lung cancer staging accuracy. This approach allows for deeper exploration and identification of critical biomarkers associated with different stages of lung cancer progression.

The main contributions of this study include: 1) proposing and implementing the MOLUNGN method, demonstrating its effectiveness in lung cancer staging; 2) validating the superior performance of MOLUNGN on multiple lung cancer datasets through comprehensive experiments; and 3) identifying and analyzing important omics features and biomarkers relevant to lung cancer staging, offering novel insights into the molecular mechanisms of lung cancer. The development and application of MOLUNGN are expected to advance the understanding of lung cancer progression, provide more accurate staging tools, and support personalized medical strategies, ultimately improving patient outcomes.

## 2 Materials and methods

### 2.1 Data sources and preprocessing

In this study, lung cancer, specifically non-small cell lung cancer (NSCLC), was selected as the primary research focus due to its clinical prevalence, accounting for approximately 85% (Lazar et al., 2024) of lung cancer cases. The richness of available clinical samples and reliability of the sources enabled a detailed exploration of biomarkers associated with lung adenocarcinoma (LUAD) and lung squamous cell carcinoma (LUSC) across different cancer stages. Initially, two primary subtypes, LUAD and LUSC, were systematically extracted from The Cancer Genome Atlas (TCGA) database.

After initial filtering, take LUAD as an example, the dataset comprised 517 LUAD samples and 1,200 case files, totaling approximately 15.3 GB of text data. For the mRNA dataset, integration was initially performed to examine intra-group correlations within omics features. Utilizing the R programming language, FPKM_unstranded values (Fragments Per Kilobase of exon model per Million mapped fragments), indicative of gene expression levels in non-strand-specific RNA-seq data, were extracted and integrated. Subsequently, these FPKM values underwent rigorous data cleaning, noise reduction, normalization, and standardization, scaling feature values to a [0,1] interval for each sample. Low-quality data exhibiting incomplete or zero expression were eliminated, refining the dataset from an initial 60,660 gene features to 14,542 high-quality genes. Dimensionality reduction was further achieved by employing a feature selection algorithm based on correlation, univariate statistical significance, and variance contribution. Following comparative analysis of multiple feature selection methods, a correlation-based approach was determined optimal, resulting in a subset of 1,000 genes for subsequent analyses.

For the LUAD miRNA dataset, preprocessing initially yielded 1,880 gene features, further refined by correlation-based feature selection to identify 512 critical miRNA features.

The DNA methylation data from LUAD, derived from the Illumina HumanMethylation450 BeadChip platform, initially included over 340,000 CpG sites and corresponding methylation values. Data preprocessing encompassed standard data matrix construction and extensive information fusion leveraging UCSC Genome Browser annotations, R-based bioinformatics processing, and standardized human gene databases. Original CpG site identifiers (e.g., “cg00000108”) were systematically replaced with adjacent gene or gene family names (e.g., “HMGN5”), ensuring consistent nomenclature across the multi-omics datasets. This step facilitated subsequent data integration, ultimately yielding 5,000 adjacent gene-associated methylation nodes.

Following separate preprocessing steps for mRNA, miRNA, and DNA methylation datasets, data fusion and cross-validation were performed based on consistent TCGA-ID sample identifiers. Samples presenting inconsistencies or incompleteness across omics datasets were excluded, leaving a refined cohort of 448 complete LUAD samples and 362 complete LUSC samples. Sample classification into normal and tumor categories for comparative analyses was conducted according to specified ranges within the 15–16 digit TCGA-ID numbers.

An identical preprocessing pipeline was applied to the LUSC dataset, and final screened omics feature summaries for both LUAD and LUSC cohorts are presented in Tables 1, 2.

**TABLE 1:** Clinical sample summary table.

Lung cancer subtypes	Multi-omics	Original cases	Preprocessed examples	Final examples
LUAD	mRNA	600	503	448
	miRNA	567	504	448
	DNA Methylation	501	453	448
LUSC	mRNA	553	495	362
	miRNA	523	474	362
	DNA Methylation	573	369	362

**TABLE 2:** Omics feature summary table.

Lung cancer subtypes	Multi-omics	Examples	Number of features
LUAD	mRNA	448	5,000
	miRNA	448	512
	DNA Methylation	448	3,856
LUSC	mRNA	362	5,000
	miRNA	362	675
	DNA Methylation	362	5,000

Clinical datasets underwent careful integration with matched omics cancer sample data. Duplicate, incomplete, or outlier data were systematically removed based on defined exclusion criteria. Outlier detection procedures and data format conversions were rigorously performed, concluding with the integration and standardization of multi-view datasets. This meticulous preprocessing preserved consistency and significantly improved dataset accuracy, thus ensuring robust and reliable experimental outcomes.

### 2.2 Method

**FIGURE 1:**
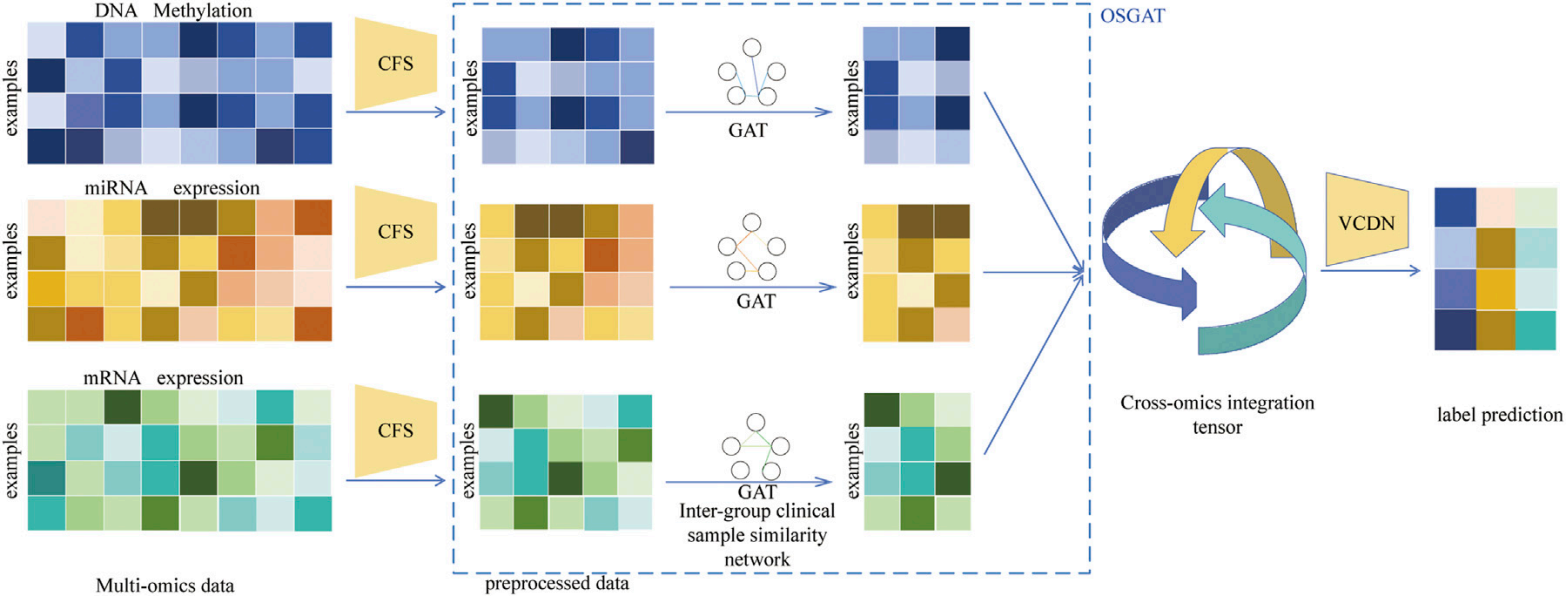
Illustration of MOLUNGN. MOLUNGN combines OSGAT for omics-specific features learning and MOVCDN for multi-omics integration. For concise illustration, an example of one patient is chosen to demonstrate the MOVCDN component for multi-omics integration after omics-specific learning. Pre-processing is first performed on each omics data type to remove noise and redundant features. OSGAT learns class prediction using omics features and the corresponding patient similarity network generated from the omics data. Cross-omics discovery tensor is calculated from initial predictions from OSGAT and forwarded to MOVCDN for final prediction. MOLUNGN is an end-to-end model and all networks are trained jointly.

This section describes the MOLUNGN model for cancer case classification. Figure 1 illustrates the MOLUNGN framework, composed primarily of two modules: omics-specific feature learning and initial label prediction within each omics group, followed by high-dimensional feature fusion and final label prediction across omics groups. Initially, in the Omics-Specific Graph Attention (OSGAT) module, three fully connected layers and the Correlation-based Feature Selection (CFS) method are employed independently for each omics dataset to reduce dimensionality and establish patient correlations for omics-specific feature extraction. Subsequently, these learned feature vectors are further processed by an enhanced Graph Attention Network (GAT) to derive initial category labels.

Following the generation of initial label predictions from each omics dataset, the Multi-Omics View Correlation Discovery Network (MOVCDN) is applied to integrate these predictions, thus significantly enhancing overall multi-omics classification accuracy. The MOLUNGN model, designed as an end-to-end approach, leverages feature fusion within a high-dimensional space to comprehensively capture correlations across multiple omics datasets, effectively achieving accurate cancer staging and classification.

As detailed in Figure 1, for each omics dataset, the Graph Attentional Layer is first used $F^{m}=ReLU\left(F^{m}\right)$ as an activation function to reduce the dimension of the original data. Additionally, a normalization layer is incorporated to mitigate potential overfitting issues. Within the OSGAT module, the dimension-reduced features from each omics dataset traverse k independent graph convolutional layers, with k dependent on the number of omics data types utilized. Each graph convolutional layer initializes the weight matrix using the Xavier initialization method (Glorot and Bengio, 2010), multiplying the feature matrix X with the weight matrix W to generate robust support vectors. This approach obviates manual specification of feature weights, ensuring reliable and consistent classification outcomes through model training.

The initial prediction label distributions, obtained via the graph attention mechanism, subsequently inform the construction of a cross-omics discovery tensor, elucidating relationships between omics labels. This tensor is reshaped into a vector and processed through two fully connected layers within MOVCDN, employing LeakyReLU as the activation function. The final classification prediction is produced through the softmax() function.

Overall, the MOLUNGN model integrates advanced graph neural network techniques to robustly and accurately classify cancer cases by reducing the complexity of multi-omics data while effectively capturing intricate relationships and interactions inherent within the datasets. Consequently, MOLUNGN demonstrates superior predictive performance and yields highly reliable classification outcomes.

#### 2.2.1 Feature initial selection

Generally, deep learning models inherently possess the capability to learn relevant features autonomously through iterative optimization of neural network weights, obviating the explicit need for separate feature selection. Nevertheless, due to the inherent ‘big p, small n' paradigm prevalent in multi-omics data (Diao and Vidyashankar, 2013), characterized by an excessive number of features (p) relative to the limited number of samples (n), deep learning approaches often encounter challenges such as unstable training and overfitting. These conditions degrade model generalizability and predictive accuracy, necessitating the integration of explicit feature selection methods (Chen et al., 2020).

Feature selection methods offer considerable advantages, notably removing irrelevant or redundant gene features, thus enhancing model performance, computational efficiency, and interpretability. The current feature gene selection methods applied to mRNA datasets include: selecting highly variable genes (Chen et al., 2016), minimizing redundancy in gene selection (Deng et al., 2023), and utilizing co-expressed gene networks (Zhang and Wong, 2022), etc. In this study, correlation-based feature selection (Gopika and ME, 2018) (CFS) was specifically chosen due to its superior capability to assess complex interactions and nonlinear dependencies between features. Unlike simpler algorithms (e.g., univariate filtering or variance thresholding), CFS comprehensively evaluates feature interactions, effectively reducing the confounding impact of redundant or irrelevant genes, thereby improving the accuracy and reliability of selected features.

In this research, CFS was independently applied to each omics dataset, selecting approximately 25% of the original features deemed most informative. Post-selection, additional data cleaning and rigorous standardization were applied before serving as inputs to the deep neural network model. Furthermore, an upper threshold was set to limit the number of features to a maximum of 5,000 per omics dataset, effectively balancing computational feasibility and robust predictive accuracy.

#### 2.2.2 Single multi-omics feature learning based on OSGAT

In the MOLUNGN model, Graph Attention Networks (GAT) are specifically utilized for individualized learning on each omics data type via the Omics-Specific Graph Attention (OSGAT) module, effectively conducting separate classification tasks. Each patient sample is represented as a node within the patient similarity network, the goal of each network is to learn a feature function f on the graph $G=\left(V,E\right)$ , which can utilize the characteristics of each node and the relationship between the nodes described by the graph G . The graph G uses the cosine similarity between samples as the edge weights for graph construction. In the graph, each node represents a patient sample, and each edge represents the cosine similarity between samples. During the graph construction process, when mining single omics data, that is, in the OSGAT module, graphs are constructed for each type of omics data and training is conducted for their respective classification tasks. In the MOVCDN module, multimodal omics data are input into VCDN for fusion training. Consequently, the OSGAT module integrates two primary inputs:

1. A feature matrix $X\in R_{n\times d}$ , where n represents the number of nodes (patients), and d is the dimension of the initial input features.
2. A structured representation of the graph, described using an adjacency matrix $A\in R_{n\times n}$ .

Given the high-dimensional characteristics of each type of omics data, three fully connected layers were first used to reduce the dimensionality and extract important features. Let X1, X2, and X3 represent the matrices of mRNA expression levels, DNA methylation levels, and miRNA expression levels in multi-omics data, respectively. For the three fully connected layers, let $X^{m}=\left\{x_{1}^{m},...,x_{N}^{m}\right\}\in R^{N\times D_{m}}$ denote the input of the *m*-th type of omics data, and $F^{m}=\left\{f_{1}^{m},...,f_{N}^{m}\right\}\in R^{N\times d_{m}}$ represent the corresponding output, where N is the number of patients, and Dm and dm are the feature dimensions of the input and output data. Initially, utilizing the Deep Graph Library (Jin et al., 2019) (DGL) within the PyTorch deep learning framework for graph construction and operations, for each node i, the unnormalized attention scores $e_{ij}$ for all its neighboring nodes *j* are calculated. Here, for the preliminary scores of nodes *i* and *j*, this study uses equation below to compute:

$$e_{ij}=LeakyReLU\left(a^{T}\left[Wh_{i}|\left|Wh_{j}\right.\right]\right)$$

Where ℎ_*i*_ and ℎ_*j*_ represent the intrinsic representation vectors of the *i* and *j* nodes in the graph, respectively. They are formed by cascading three types of omics features: mRNA, miRNA, and DNA Methylation, after normalization, with the aim of maximally condensing cross-modal biological clues. *W* is a globally shared linear projection matrix, which compresses the heterogeneous and high-dimensional inputs into a unified latent space, making the downstream relevance measurements comparable and aggregatable in terms of dimension and scale. *a* can be regarded as a learnable “probe” vector, which performs a weighted inner product on the concatenated [*W*ℎ_i_||*W*ℎ_*j*_], thereby producing a non-normalized attention raw score for the edge (*i*,*j*).

To facilitate the comparison and induction of coefficients between different nodes, the softmax() function is used for normalization, ensuring that the sum of attention coefficients between each node and its neighboring nodes is 1, as shown in equation as follows:

$$a_{ij}=softmax_{j}\left(e_{ij}\right)=\frac{\exp\left(e_{ij}\right)}{\sum_{k\in N_{i}}\exp\left(e_{ik}\right)}$$

Based on the aggregation characteristics of attention weights, the features of neighbors are updated.

$$T^{\left(l+1\right)}=\sigma \left(\sum_{j\in N_{i}}a_{ij}T_{i}^{\prime }\right)$$

Where ${T_{i}}^{\prime }$ is the transformed feature of node *i*, which dynamically adjusts its own features through attention weights $a_{ij}$ ​.

GAT is widely used in classification learning tasks of graph structured data, and the input matrix of this model contains node structures and graph structures. Therefore, both the internal features and graph structures of the training data were used for learning the classification task.

#### 2.2.3 Multi-omics feature fusion learning based on MOVCDN

Existing deep learning-based multi-omics integration methods typically fuse features from distinct omics modalities either in the initial input or intermediate feature space (Zhang X. et al., 2021; Zhu et al., 2015). Previous research demonstrates that the View Correlation Discovery Network (VCDN) effectively captures correlations between diverse data views within the label space, particularly for tasks such as human action recognition (Wang et al., 2019). Inspired by this, Wang (Wang et al., 2020) applied VCDN to various types of omics data for biological data classification. In this study, VCDN was used to model the correlation between multi-omics data, and the module was named MOVCDN. Let $M_{j}\in R^{C\times C\times C}$ represent the cross-omics discovery tensor of the *j*-th sample. The specific calculation equation is as follows:

$$M_{j,abc}={\hat{y}}_{j,a}^{\left(1\right)}{\hat{y}}_{j,b}^{\left(2\right)}{\hat{y}}_{j,c}^{\left(3\right)}$$

Where ${\hat{y}}_{j,x}^{\left(i\right)}$ denotes the *x*th term ${\hat{y}}_{j}^{\left(i\right)}$ . Next, cross-omics found that tensor $T_{j}$ was reshaped into an $C^{3}$ -dimensional vector. Next, the vector is input into a two-layer fully connected layer with an output dimension of C for final label prediction. MOVCDN uses cross entropy loss for training, that is:

$$L_{MOVCDN}=\sum_{j=1}^{n_{tr}}L_{CE}\left(MOVSDN\left(T_{j}\right),y_{j}\right)$$

$$=\sum_{j=1}^{n_{tr}}-\log\left(\frac{e^{MOVCDN\left(T_{j}\right)\cdot y_{j}}}{\sum_{i=1}^{k}e^{MOVCDN{\left(T_{j}\right)}_{k}}}\right)$$

Where $L_{CE}$ is the cross entropy loss function, and $MOVCDN{\left(T_{j}\right)}_{k}$ is the *k*-th element in the vector $MOVCDN\left(T_{j}\right)\in R^{C}$ .

The total loss function of MOLUNGN can be expressed as:

$$L=\sum_{i=1}^{3}L_{C}^{\left(i\right)}+ðL_{MOVCDN}$$

Where $L_{C}^{\left(i\right)}$ sets a separate supervision signal for the *i*-th omics branch (*i*∈{1,2,3}, corresponding to mRNA, miRNA, and DNA Methylation respectively) to ensure that each modality has independent discriminative capability before fusion. ð is the trade-off parameter between the classification loss of each omics and the final classification loss of MOVCDN. In this article, set ð=1 . In summary, MOLUNGN is an end-to-end model that aims to jointly explore the correlation of cases in the internal characteristics of omics information and the correlation of cross-omics in the label space.

### 2.3 Experiment

#### 2.3.1 Contrast model

In this experiment, MOLUNGN was rigorously compared with 10 baseline classifiers, including KNN, Support Vector Machine (SVM), LASSO, Random Forest (RF), XGBoost, and the improved LUNGGCN model. For the KNN classifier, the parameter K was set to 5. The SVM classifier utilized a kernel function to project data into higher-dimensional spaces, facilitating the classification of complex datasets. In LASSO, a separate model was trained for each class, with final predictions determined by selecting the label with the highest predicted probability. The RF classifier employed multiple decision trees constructed from diverse data subsets. Models such as KNN, SVM, LASSO, and RF were trained by concatenating multi-omics data, predominantly using the Scikit-learn library (Kramer and Kramer, 2016) with default parameter settings. The MOLUNGN and improved LUNGGCN models were implemented using the PyTorch framework, maintaining consistent parameter settings across both models. For evaluation, 70% of samples were randomly assigned as the training set, with the remaining 30% as the test set. This partitioning procedure was repeated ten times to create different randomized train-test groups, and performance metrics across these ten experiments were comprehensively reported.

#### 2.3.2 Parameter setting

The parameters used in the models during the experiment are shown in Table 3.

**TABLE 3:** Model parameter settings.

Parameter	MOLUNGN	MOGONET	LUNGGCN
Layer	Graph Attentional Layer	Graph Convolution Layer	Graph Attentional Layer
Num_epochs	200	200	200
Num_view	3	3	3
Num_class	5	5	5
Num_heads	32	-	32
Out_dims	2	2	2
Hidden_dims	16	16	16
Learning rate	0.008	0.008	0.008
Vcdn_features	128	128	-

#### 2.3.3 Evaluating indicatorl

(1) Accuracy rate

$$Accuracy=\frac{TP+TN}{TP+TN+FP+FN}$$

The evaluation index represents the correct proportion of the model in all predictions. Among them, TP (True Positive): True positive, that is, the number of positive classes correctly predicted by the model; TN (True Negative):True negative, that is, the number of negative classes correctly predicted by the model; FP(False Positive):False positive, that is, the number of positive classes that the model incorrectly predicts; FN(False Negative):false negative, that is, the number of negative classes that the model incorrectly predicts.

(2) Weighted recall rate

$$Recall_{weighted}=\sum_{i=1}^{N}\left(\frac{{support}_{i}}{total\_support}\times \frac{{TP}_{i}}{{TP}_{i}+{FN}_{i}}\right)$$

Recall_weighted is an important indicator used to measure the performance of classification models in machine learning. It mainly focuses on the recall performance of the model on different categories, and comprehensively considers the number of samples in each category. It provides a single score to evaluate the recall performance of the model in multi-classification problems, especially in the case of unbalanced category distribution. The recall rate is calculated for each category: First, the recall rate (Recall) of each category is calculated, that is, the proportion of the number of samples correctly predicted as positive cases in the category to the total number of actual positive cases in the category. Calculate the weighted average recall rate: then, multiply the recall rate of each category by the proportion of the category in the total sample (weight), and then sum the weighted recall rates to obtain the final ' Recall_weighted ' score.

(3) F1 score

Typically, the calculation equation for the F1 score of the *i*-th category is the harmonic mean of the precision and recall rates, defined as follows:

$${F1}_{i}=2\times \frac{{Precision}_{i}\times {Recall}_{i}}{{Precision}_{i}+{Recall}_{i}}$$

This evaluation index is an important indicator used to measure the performance of classification models in machine learning. It comprehensively considers the accuracy rate and recall rate, and provides a single score to evaluate the accuracy of the model. The F1 score can be subdivided into F1_macro and F1_weighted according to the actual data of the model and the feedback of the results.

$$F1\_macro=\frac{1}{N}\times \sum_{i=1}^{N}\left({F1}_{i}\right)$$

F1_macro calculates F1 scores for each category, and then calculates the arithmetic mean of these F1 scores. This approach treats all categories equally, regardless of the importance of the category or the number of samples. That is to say, each category has the same contribution to the final F1_macro score.

$$F1\_weighted=\sum_{i=1}^{N}\left(\frac{support}{tota\_support}\times {F1}_{i}\right)$$

F1_weighted calculates F1 scores for each category, but when calculating the average, the number of samples in each category is used as the weight. This means that the category with a larger number of samples has a greater impact on the final F1_weighted score. This method takes into account the importance of categories, especially in the case of uneven distribution of categories, and can give a more balanced performance index.

### 2.4 Results and discussion

#### 2.4.1 Comparison with other state-of-the-art algorithms

**FIGURE 2:**
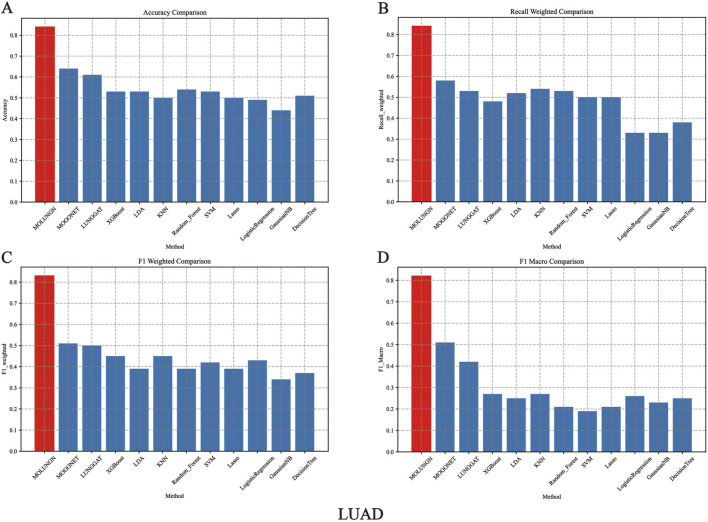
The performance comparison of algorithms under LUAD data set. **(A)** Accuracy comparison. **(B)** Recall weighted comparison. **(C)** F1 weighted comparison. **(D)** F1 macro comparison.

**FIGURE 3:**
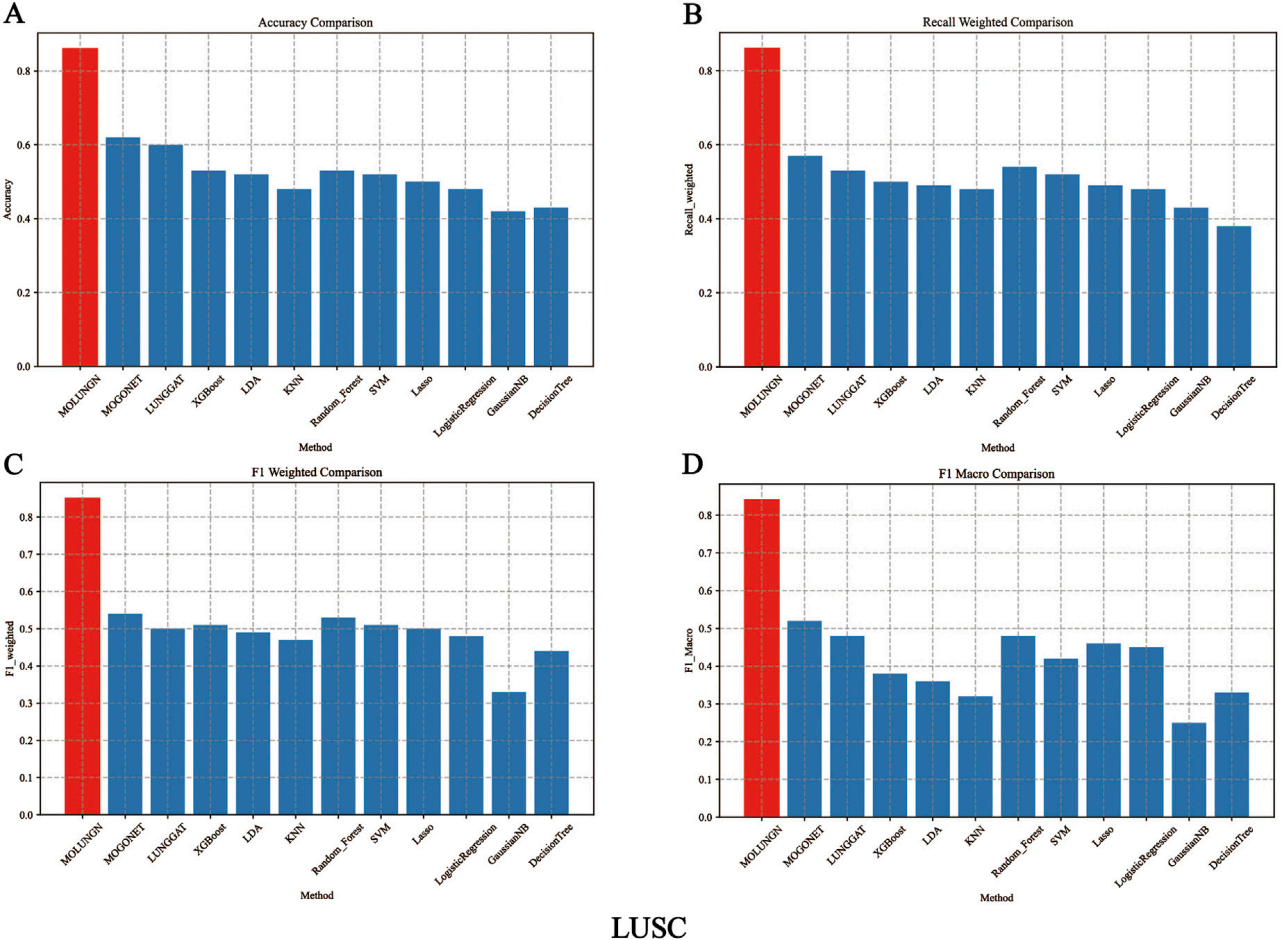
The performance comparison of algorithms under LUSC data set. **(A)** Accuracy comparison. **(B)** Recall weighted comparison. **(C)** F1 weighted comparison. **(D)** F1 macro comparison.

In this experiment, the performance of the proposed MOLUNGN method was comprehensively compared with eleven baseline classifiers, including K-nearest neighbors (KNN), support vector machine (SVM), LASSO, random forest (RF), XGBoost, linear discriminant analysis (LDA), decision tree, logistic regression (LR), and the improved LUNGGCN model. The comparative analysis of these algorithms is detailed in Table 4 and visually represented in Figures 2, 3.

**TABLE 4:** Algorithm performance comparison.

Dataset	Method	ACC	Recall_weighted	F1_weighted	F1 macro
LUAD	MOLUNGN	0.84	0.84	0.83	0.82
	MOGONET	0.64	0.58	0.51	0.51
	LUNGGAT	0.61	0.53	0.50	0.42
	XGBoost	0.53	0.48	0.45	0.27
	LDA	0.53	0.43	0.39	0.25
	KNN	0.50	0.52	0.45	0.27
	Random_Forest	0.54	0.54	0.39	0.21
	SVM	0.53	0.53	0.42	0.19
	Lasso	0.50	0.50	0.39	0.21
	LogisticRegression	0.49	0.50	0.43	0.26
	GaussianNB	0.44	0.33	0.34	0.23
	DecisionTree	0.51	0.38	0.37	0.25
LUSC	MOLUNGN	0.86	0.86	0.85	0.84
	MOGONET	0.62	0.57	0.54	0.52
	LUNGGAT	0.60	0.53	0.50	0.48
	XGBoost	0.53	0.50	0.51	0.38
	LDA	0.52	0.49	0.49	0.36
	KNN	0.48	0.48	0.47	0.32
	Random_Forest	0.53	0.54	0.53	0.48
	SVM	0.52	0.52	0.51	0.42
	Lasso	0.50	0.49	0.50	0.46
	LogisticRegression	0.48	0.48	0.48	0.45
	GaussianNB	0.42	0.43	0.33	0.25
	DecisionTree	0.43	0.38	0.44	0.33

Figures 2, 3 comprehensively illustrate the performance comparison among 12 different classifiers on the LUAD and LUSC datasets, highlighting the superior performance of the proposed MOLUNGN model across multiple evaluation metrics. Notably, both MOLUNGN and the MOGONET model implemented in this study contain two core algorithm modules: a Graph Neural Network-based core module and a Multi-omics View Correlation Discovery Network (MOVCDN) module designed specifically for multi-omics data fusion. In contrast, the remaining baseline classifiers employ only single-core algorithm modules, resulting in limited effectiveness in capturing complex multi-modal interactions.

By comparing MOLUNGN with the LUNGGAT model, it was clearly demonstrated that excluding the MOVCDN multi-modal fusion module resulted in significantly reduced classification performance. Specifically, without MOVCDN, the LUNGGAT model achieved evaluation metrics of ACC (0.60), Recall_weighted (0.53), F1_weighted (0.50), and F1_macro (0.48) on the LUSC dataset. However, incorporating the MOVCDN module dramatically enhanced these metrics, increasing ACC to 0.86, Recall_weighted to 0.86, F1_weighted to 0.85, and F1_macro to 0.84, thereby affirming the superior efficacy of multi-omics integration in practical biomedical applications.

Moreover, the comparative analysis between MOLUNGN and MOGONET further emphasized the advantage of the Graph Attention Network (GAT) core module over traditional Graph Convolutional Networks (GCN) in processing large-scale systems biology data. Specifically, the GCN-based MOGONET model exhibited lower performance (ACC: 0.62, Recall_weighted: 0.57, F1_weighted: 0.54, F1_macro: 0.52) compared to the GAT-based MOLUNGN model. These observations indicate that the graph attention mechanism of the GAT module demonstrates superior feature extraction and information fusion capabilities.

In summary, experimental results robustly illustrate that MOLUNGN significantly enhances the efficacy of biomedical data mining associated with lung cancer by integrating advanced graph attention mechanisms and multi-modal omics data fusion, thereby demonstrating substantial potential for clinical translation and future biomarker discovery.

#### 2.4.2 Ablation study

**FIGURE 4:**
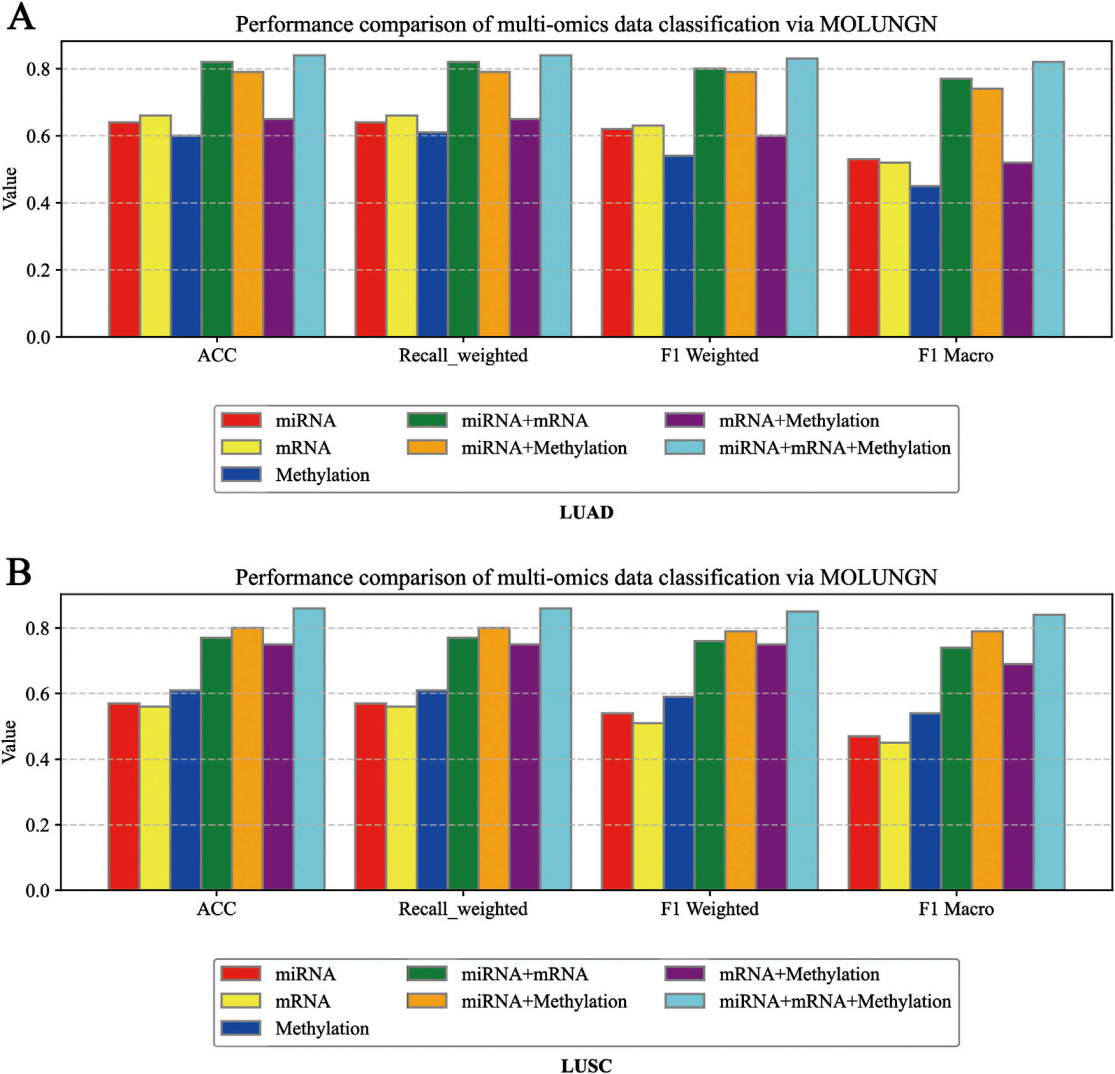
**(A)** Ablation experiment diagram under LUAD dataset; **(B)** Ablation experiment diagram under LUSC dataset Ablation experiment diagram under LUAD dataset.

Through comprehensive ablation experiments, we systematically evaluated the impact of varying quantities of omics-specific features, different training set partitioning methods, and multiple combinations of omics data on the performance of the proposed MOLUNGN model, with particular attention to assessing the significance of multi-omics integration. To thoroughly explore the contribution of individual omics features and their combinations, seven different omics integration strategies were designed and examined, namely,: mRNA alone, miRNA alone, DNA methylation alone, mRNA combined with DNA methylation, mRNA combined with miRNA, miRNA combined with DNA methylation, and the comprehensive integration of all three omics modalities (mRNA + DNA methylation + miRNA). Figure 4 presents detailed comparisons regarding the performance of these omics data combinations.

Experimental findings indicated that the single omics dataset based solely on mRNA expression demonstrated the lowest predictive performance, attributed to its limited number of features, minimal complexity in the graph structure, and reduced node-level biological information. Conversely, dual omics combinations, such as mRNA combined with miRNA or DNA methylation, consistently resulted in significantly improved performance across various evaluation metrics. The continuous enhancement of the model’s performance indicators when integrating multi-modal data from dual to triple omics further confirmed that multi-modal fusion substantially enhances model performance.

Figures 4A,B explicitly compare model performance across various omics perspectives, employing three evaluation indicators to analyze how different types of omics data influence model efficacy. In these figures, distinct colored histograms represent different omics data combinations utilized in the ablation experiments.

In the ablation analysis, one or several types of omics data were systematically removed to observe corresponding changes in model performance. The results consistently demonstrated that omitting any individual omics dataset led to a notable decline in classification performance, underscoring the crucial importance of integrating multi-omics data to achieve optimal classification accuracy. Specifically, models relying on only a single type of omics data exhibited relatively lower predictive accuracy; conversely, integrating multiple types of omics data allowed the model to capture a richer set of potential features and associations, thus providing more accurate and reliable classification results. Additionally, by examining the performance diagrams of the single-omics models versus multi-omics fusion models, we observed that the effectiveness of individual omics modalities significantly impacts the performance achieved through multi-omics integration, confirming the model’s ability to effectively incorporate features from multiple omics dimensions.

Moreover, although increasing the feature dimensionality inevitably raises the computational complexity, the availability of richer omics data significantly enhances the model’s discriminative capabilities among different sample categories. These findings further substantiate the essential role of strategic feature selection and multi-omics integration in biomedical data analysis. By retaining the most relevant and informative omics features, the model can comprehensively capture inter-sample differences, thus markedly improving classification accuracy.

In conclusion, the ablation experiments clearly illustrate that increasing the number of features within individual omics datasets and integrating a greater diversity of omics modalities significantly enhances node-level information richness and graph complexity, thereby substantially improving overall model performance. Incorporating multi-modal data allows more comprehensive extraction and utilization of biological information from multiple levels and perspectives, not only enhancing classification accuracy but also providing robust support for biomarker discovery and disease mechanism research.

In summary, the ablation studies yielded the following key conclusions.

(1) The informational content and number of features in a single omics dataset significantly influence model performance.
(2) Multi-omics data integration markedly improves classification accuracy and overall model performance.
(3) Different omics modalities provide complementary biological insights; thus, their integration comprehensively captures the biological characteristics of tumors.
(4) Increasing the number and diversity of features increases model complexity but simultaneously enhances the model’s node information content and classification capabilities.
(5) Ablation experiments provide strong empirical support for the necessity of multi-omics integration and the critical importance of effective feature selection strategies.

#### 2.4.3 Identify the key biomarkers by MOLUNGN model

In the final section of this study, multimodal omics datasets were systematically integrated with corresponding clinical annotations derived from publicly available lung cancer case samples. Utilizing the clinically recognized TNM cancer staging system (SchrlSder et al., 1992), each patient’s cancer stage was meticulously extracted, thereby enabling the integration and categorization of samples according to their clinical stages. The MOLUNGN model was subsequently employed to effectively classify the integrated multi-omics data across various cancer stages and to robustly identify stage-specific biomarkers pivotal for disease progression. To achieve a nuanced understanding of the biomarkers associated with distinct stage transitions, this study converted the complex multi-stage classification task into multiple binary classification scenarios. For instance, in the LUAD dataset, samples from stage I and stage II were specifically isolated and analyzed within the MOLUNGN framework to elucidate biomarkers indicative of the biological transformation occurring between these two stages. The LUAD dataset comprised stage I through stage IV samples, while the LUSC dataset encompassed stages I through III.

By synthesizing multiple data modalities, including mRNA expression, miRNA expression, and DNA methylation profiles, MOLUNGN provides a multi-level, comprehensive approach for investigating critical driver genes and biomarkers implicated in lung cancer progression. From the systems biology perspective, this integrative methodology facilitates deeper insights into the molecular mechanisms underlying lung cancer initiation, progression, and metastasis. Moreover, the identified biomarkers and corresponding genes were systematically organized and incorporated into the complex network model of “Traditional Chinese Medicine (TCM)–Target–Disease,” further enriching data-driven insights for exploring biological mechanisms and potential therapeutic targets in traditional Chinese medicine-based treatments for lung cancer.

**FIGURE 5:**
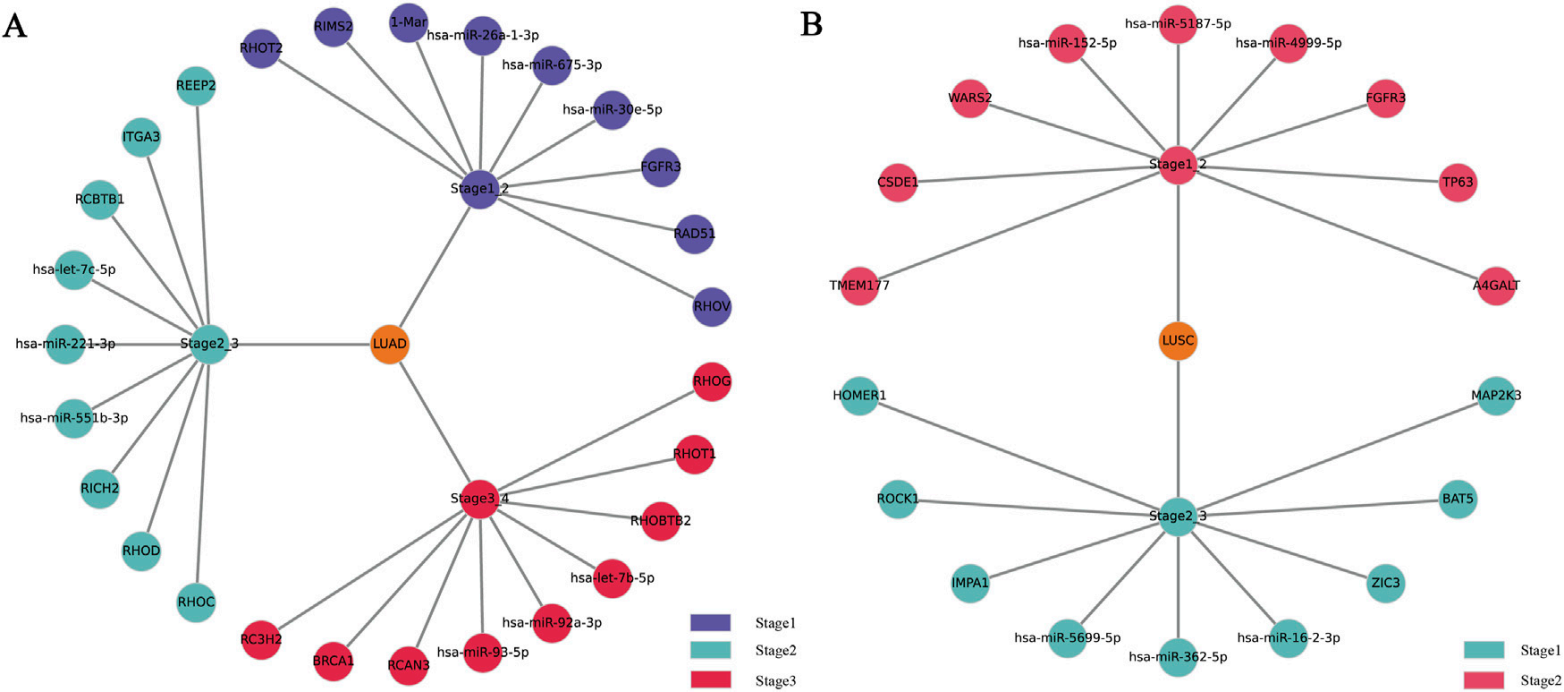
The figure includes a partial display of the important biomarkers discovered during the progression and stage transition of **(A)** LUAD and **(B)** LUSC.

Further in-depth data mining identified key biomarkers distinctly associated with specific lung cancer stages, the screening criterion for the top biomarkers is to select biomarkers in descending order based on the characteristic attention weight coefficient, as explicitly summarized in Table 5 and visually represented in Figure 5. Notably, previous studies conducted by Wang (Wang et al., 2022), Zhang (Zhang D. et al., 2021), and Shepelev (Shepelev and Korobko, 2013) have demonstrated that the RHOV gene, a prominent member of the Rho family of GTPases, significantly regulates NSCLC gene expression profiles. In particular, RHOV is notably overexpressed in lung adenocarcinoma (LUAD) tissues, where it strongly correlates with patient prognosis and survival outcomes. Mechanistically, RHOV actively promotes LUAD cell proliferation, migration, and invasive capabilities through activation of the JNK/c-Jun signaling pathway. Additionally, hsa-miR-30e-5p29(Xu et al., 2018), another critical biomarker identified, exerts tumor-suppressive effects on NSCLC via modulation of the USP22-mediated Sirt1/JAK/STAT3 signaling pathway. Indeed, members of the miRNA-30 family (Yu and Sui, 2020) have been widely recognized for their regulatory significance in NSCLC pathogenesis.

**TABLE 5:** Important omics biomarkers identified at different stages in LUAD and LUSC dataset.

Dataset	Stage	Omics type	Top import biomarkers
LUAD	StageⅠ、Ⅱ	mRNA expression	RHOV、RAD51、FGFR3
		miRNA expression	hsa-miR-30e-5p、hsa-miR-675-3p、hsa-miR-26a-1-3p
		DNA methylation	1-Mar、RIMS2、RHOT2
	StageⅡ、Ⅲ	mRNA expression	REEP2、ITGA3、RCBTB1
		miRNA expression	hsa-let-7c-5p、hsa-miR-221-3p、hsa-miR-551b-3p
		DNA methylation	RICH2、RHOD、RHOC
	StageⅢ、Ⅳ	mRNA expression	RC3H2、BRCA1、RCAN3
		miRNA expression	hsa-miR-93-5p、hsa-miR-92a-3p、hsa-let-7b-5p
		DNA methylation	RHOBTB2、RHOT1、RHOG
LUSC	StageⅠ、Ⅱ	mRNA expression	A4GALT、TP63、FGFR3
		miRNA expression	hsa-miR-4999-5p、hsa-miR-5187-5p、hsa-miR-152-5p
		DNA methylation	WARS2、CSDE1、TMEM177
	StageⅡ、Ⅲ	mRNA expression	HOMER1、ROCK1、IMPA1
		miRNA expression	hsa-miR-5699-5p、hsa-miR-362-5p、hsa-miR-16-2-3p
		DNA methylation	ZIC3、BAT5、MAP2K3

Furthermore, our analysis identified the BRCA1 gene as a critical biomarker with notably diminished mRNA and protein expression observed in lung adenocarcinoma and squamous cell carcinoma, respectively. Promoter hypermethylation has been established as a principal regulatory mechanism driving the aberrant expression of BRCA1(Lee et al., 2007), thereby contributing substantially to NSCLC pathogenesis. Additionally, the FGFR3 gene (Liao et al., 2013), a member of the transmembrane receptor tyrosine kinase (RTK) superfamily encoding single-chain glycoproteins, emerged as another significant biomarker. FGFR3 is known to exert pronounced inhibitory effects in lung cancer, particularly lung squamous cell carcinoma, and thus represents a promising therapeutic target for NSCLC treatment.

In addition, when a new case enters the prediction process, it is necessary to first obtain its mRNA transcript abundance, miRNA expression profile, and DNA methylation level data. After quality control, imputation of missing sites, batch effect correction, and Z-score normalization of the raw read segments, the three types of features are cascaded in the established order of the training phase into a unified numerical vector, and directly input into the graph attention network that has completed offline training. Since GAT belongs to the inductive learning paradigm, focusing on the generalization mapping of “node features to class labels” rather than relying on static full graph topology, there is no need to reconstruct the adjacency matrix during the inference phase to output the posterior probability of lung cancer staging or molecular subtypes; if it is subsequently desired to improve model performance, new samples can be incorporated into the training set for full retraining or progressive fine-tuning, while the timeliness and stability of routine clinical inference links are not affected.

Collectively, comprehensive pharmacological literature review and comparative analyses demonstrate remarkable consistency between MOLUNGN model predictions and empirical outcomes from experimental studies involving cellular and animal models, further validating the scientific rigor, reliability, and translational potential of the proposed MOLUNGN framework in lung cancer biomarker discovery and mechanistic exploration.

### 2.5 Conclusion

In this paper, we proposed a novel multi-omics integrated graph deep learning framework named MOLUNGN, explicitly designed for the precise staging and classification of lung cancer using comprehensive biomedical datasets. MOLUNGN integrates specialized omics-specific prediction modules based on Graph Attention Networks (GAT), effectively capturing intra-omics characteristics and leveraging intrinsic patient similarities within individual omics datasets. Furthermore, a Multi-Omics View Correlation Discovery Network (MOVCDN) was developed to model complex cross-omics correlations in the high-level label space, substantially enhancing classification accuracy and robustness.

Utilizing publicly available lung cancer datasets and clinical annotations, our MOLUNGN model demonstrated superior performance compared to state-of-the-art baseline methods. Specifically, MOLUNGN achieved impressive results on the LUAD dataset, with accuracy (ACC) of 0.86, weighted recall (Recall_weighted) of 0.86, weighted F1-score (F1_weighted) of 0.85, and macro F1-score (F1_macro) of 0.84. Similar performance enhancements were observed on the LUSC dataset, with ACC of 0.86, Recall_weighted of 0.86, F1_weighted of 0.85, and F1_macro of 0.84. Ablation studies further verified the significant contributions of the GAT and MOVCDN modules and demonstrated the clear advantages of multi-omics data integration over single-omics approaches.

Moreover, the MOLUNGN model successfully identified critical biomarkers from multi-modal omics datasets closely associated with distinct stages of lung cancer progression. These biomarkers, such as RHOV, hsa-miR-30e-5p, and RICH2, have demonstrated significant regulatory roles in the mechanisms underlying lung adenocarcinoma and squamous cell carcinoma. Additionally, the identified stage-specific biomarkers were systematically integrated into the “Traditional Chinese Medicine-Target-Disease” network model, providing an enriched biological data framework and valuable methodological references for exploring the comprehensive biological mechanisms of traditional Chinese medicine in lung cancer therapy.

Future research will further extend the MOLUNGN model by incorporating additional multimodal clinical data types, including patient behavioral information and medical imaging, aiming to construct an even more comprehensive analytical framework for understanding disease mechanisms, enhancing predictive accuracy, and advancing personalized medicine.

## Data Availability

The source code to reproduce the results are available at https://github.com/labnjucm/MOLUNGN. Further information can be obtained from the corresponding author.
